# *Aedes albopictus* production in urban stormwater catch basins and manhole chambers of downtown Shanghai, China

**DOI:** 10.1371/journal.pone.0201607

**Published:** 2018-08-09

**Authors:** Qiang Gao, Fei Wang, Xihong Lv, Hui Cao, Fei Su, Jianjun Zhou, Peien Leng

**Affiliations:** 1 Department of Vector Prevention, Shanghai Huangpu Center for Disease Control & Prevention, Shanghai, People’s Republic of China; 2 Department of Vector Prevention, Shanghai Hongkou Center for Disease Control & Prevention, Shanghai, People’s Republic of China; 3 Department of Vector Prevention, Shanghai Songjiang Center for Disease Control & Prevention, Shanghai, People’s Republic of China; 4 Department of Vector Prevention, Shanghai municipal Center for Disease Control & Prevention, Shanghai, People’s Republic of China; University of Texas Medical Branch at Galveston, UNITED STATES

## Abstract

**Background:**

The near-surface urban drainage system in Shanghai is highly complex, with hundreds of thousands of catch basins (CBs) and manhole chambers (MCs). Comparatively little is known about the breeding of mosquitoes in this vast system, especially for the locally predominant species *Aedes albopictus*. A cross-sectional mosquito sampling study was conducted from late July to early August of 2017 using 539 CBs and 309 MCs located in 10 communities of downtown Shanghai. We measured the water-holding status of the drainage systems and density of mosquito larvae. Mosquito species were examined on site and in the laboratory later.

**Results:**

The CBs were characterized by a lower percentage of standing water compared to MCs (47.0% vs. 79.9%, respectively; X^2^ = 76.407, P<0.001), but CBs contained a higher percentage of stagnant water percent than MCs (45.2% vs. 35.3%, respectively; X^2^ = 11.465, P = 0.001). There were exclusively two species of mosquito larvae found in the drainage systems, *Ae*. *albopictus* and *Culex pipiens* complex. Compared with MCs, the structures of CBs were more conducive to larval production and yielded more larvae-positive samples (43.4% vs 14.2%, X^2^ = 53.136, P<0.001) and higher larval density (8.23 vs. 4.09 per dipper, t = 3.287, P = 0.001). *Aedes albopictus* was the predominant species in CBs, with a constituent ratio of 71.7%. Regarding structures with different features in different locations, CBs that had a vertical grate with an unsealed lid and MCs with plastic composite covers were the most favorable types for mosquito breeding, and residential neighborhoods yielded the highest number of *Ae*. *albopictus*.

**Conclusion:**

*Aedes albopictus* was the predominant species in both CBs and stormwater MCs, especially in residential neighborhoods. CBs, particularly those with vertical grates, were a major source of mosquito production in downtown Shanghai. MCs featured more running water and fewer larvae by percentage, and few larvae were found in Sewage MCs. However, due to the tremendous baseline amount, MCs were still an important breeding source of mosquitoes. We suggest that *Aedes* control in Shanghai should focus on CBs or other potential larvae habitats in and around residential neighborhoods. The use of permeable materials and completely sealed covers should be adopted in the construction of CBs and MCs henceforth.

## Background

The Asian tiger mosquito *Aedes albopictus* (Skuse, 1894) is the most concern of nuisance and perhaps the most medically important vector species in Shanghai[1] due to its predominant populations, highly anthropophilic in host preference, and potential role in transmitting dengue, chikungunya and Zika viruses to humans[2, 3]. The public health threat posed by *Ae*. *albopictus* has made it a top priority for vector control efforts in Shanghai. Mosquito control directed at immature stages in standing water rather than at adults is most effective [4, 5], and thus identifying larval breeding habitats is paramount. Female *Ae*. *albopictus* prefer to lay eggs in a variety of small natural and artificial containers, e.g., in tree-holes, flower pots, tin cans, water jars, metal and wooden buckets or drums, broken glass bottles, and discarded motor vehicle tires [6]. However, when the number of small containers is reduced due to urbanization and effective sanitation campaigns, moderate-sized water-holding containers such as catch basins (CBs) and manhole chambers (MCs) of near-surface urban drainage systems may become potential alternative breeding habitats for *Ae*. *albopictus* in downtown areas [7], and these water-holding structures are often overlooked in routine mosquito monitoring because of the difficulty in accesssing the structure [8].

Shanghai is the largest metropolis in East China (population > 24 million) with a highly complex near-surface drainage system that consists of hundreds of thousands of stormwater CBs and MCs. These structures were designed and constructed with multiple features or styles in different regions during the past decades. The below-surface portions of these devices are characterized by stable microclimatic conditions, offering potentially ideal habitats for breeding of immature mosquitoes and resting of adults domestic and peridomestic mosquitoes [9]. By the conservative estimates by Su et al[9], even if only 10% of the system supports mosquito production, the system would aggregately constitute the largest mosquito breeding sites in the urban areas.

Numerous studies have been conducted on mosquito breeding in stormwater infrastructures for catchment and conveyance of runoff in North and South America[7, 9–14], Europe[15, 16], Oceania and South Asia [17, 18], all of which indicated that stormwater treatment devices like CBs served as major development and resting sites for anthropophilic and zoophilic mosquitoes in urban environments. However, due to differences in usage, structural features, and locations, larval abundance varied significantly across these structures at a fine spatial scale [5]. In China, studies involving mosquito production in these drainage systems were lacking, moreover, in the background of most studies suggesting stormwater drains were more conducive for the breeding of *Culex* species mosquitoes, little is known about *Aedes* species mosquitoes in sub-surface drainage systems in areas dominated by *Ae*. *albopictus*, such as the urban areas of Shanghai.

To provide a basis for the management of mosquitoes (especially for *Ae*. *albopictus*) in these vast systems in Shanghai and its surrounding areas, detailed information is needed including water-holding status and mosquito species composition in these diverse structures. Here we report a cross-sectional survey of mosquito production from 858 CBs and MCs located in 10 communities in downtown Shanghai. Our goal is to elucidate the association between larval abundance and features or locations of these devices and to further illuminate which types or designs of these structure were more conducive to *Ae*. *albopictus* production in urban areas.

## Materials and methods

### Area and timing of study

**Fig 1:**
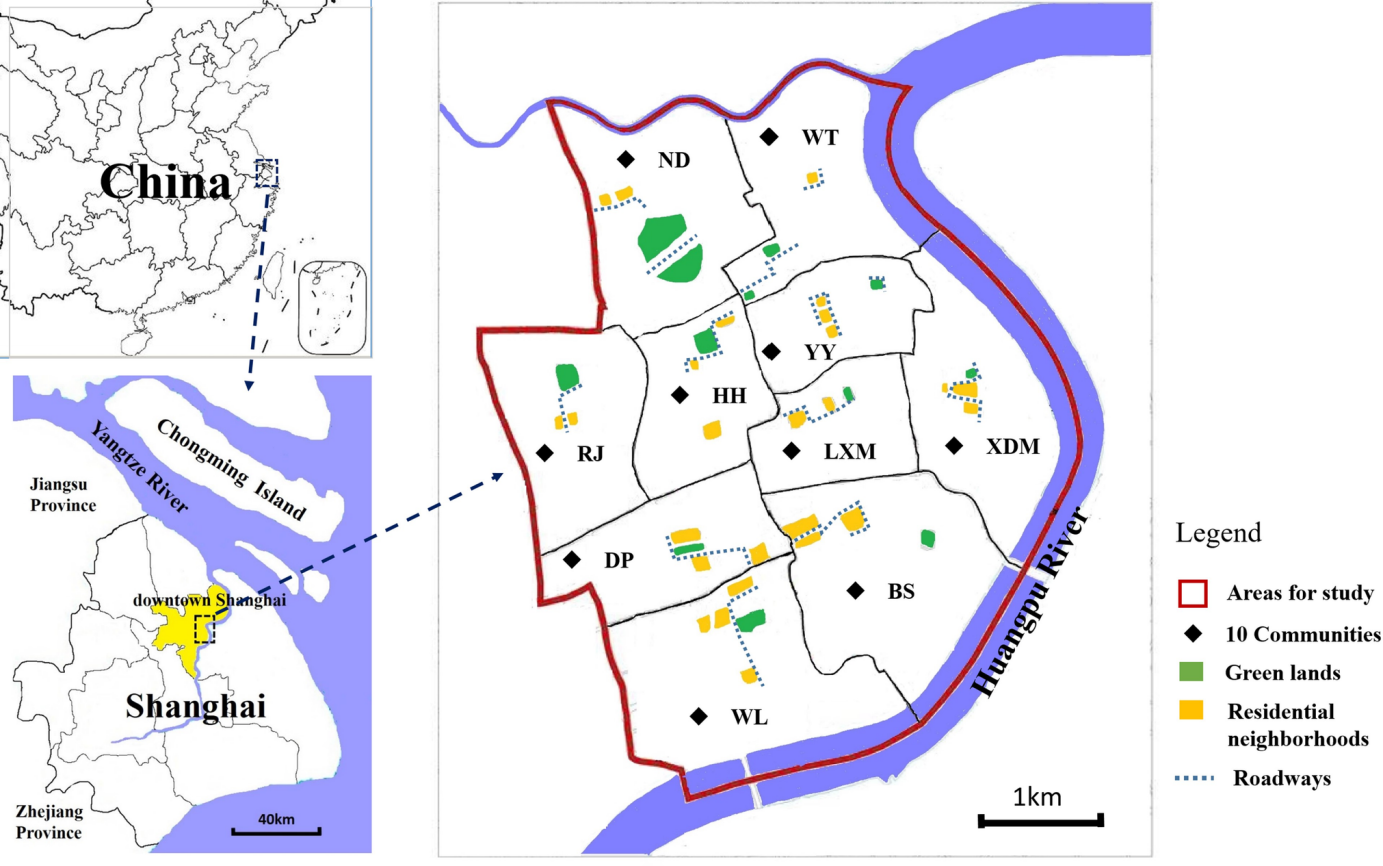
Areas and 10 communities sampled in the study (note: this figure was recreated using an open source map from https://commons.wikimedia.org/wiki/Atlas_of_the_world).

The studies were carried out in downtown areas of Shanghai (population ~650,000; 31°13′N, 121°27′E and 3.5m above sea level), comprising 10 communities. Each community included three environment types for mosquito sampling: a) parklands, b) residential neighborhoods and c) streets or roadways (Fig 1). The approximate cross-sectional study was undertaken in late July and early August (last for < 20 days), which was also the hottest season of the year (mean day-temperature ranged from 28°C~35°C). We chose the most ubiquitous two structure types of sub-surface drainage system in downtown Shanghai as study objects, these were CBs and MCs. All collections of mosquito samples were done on public land.

#### Catch basins (CBs)

**Fig 2:**
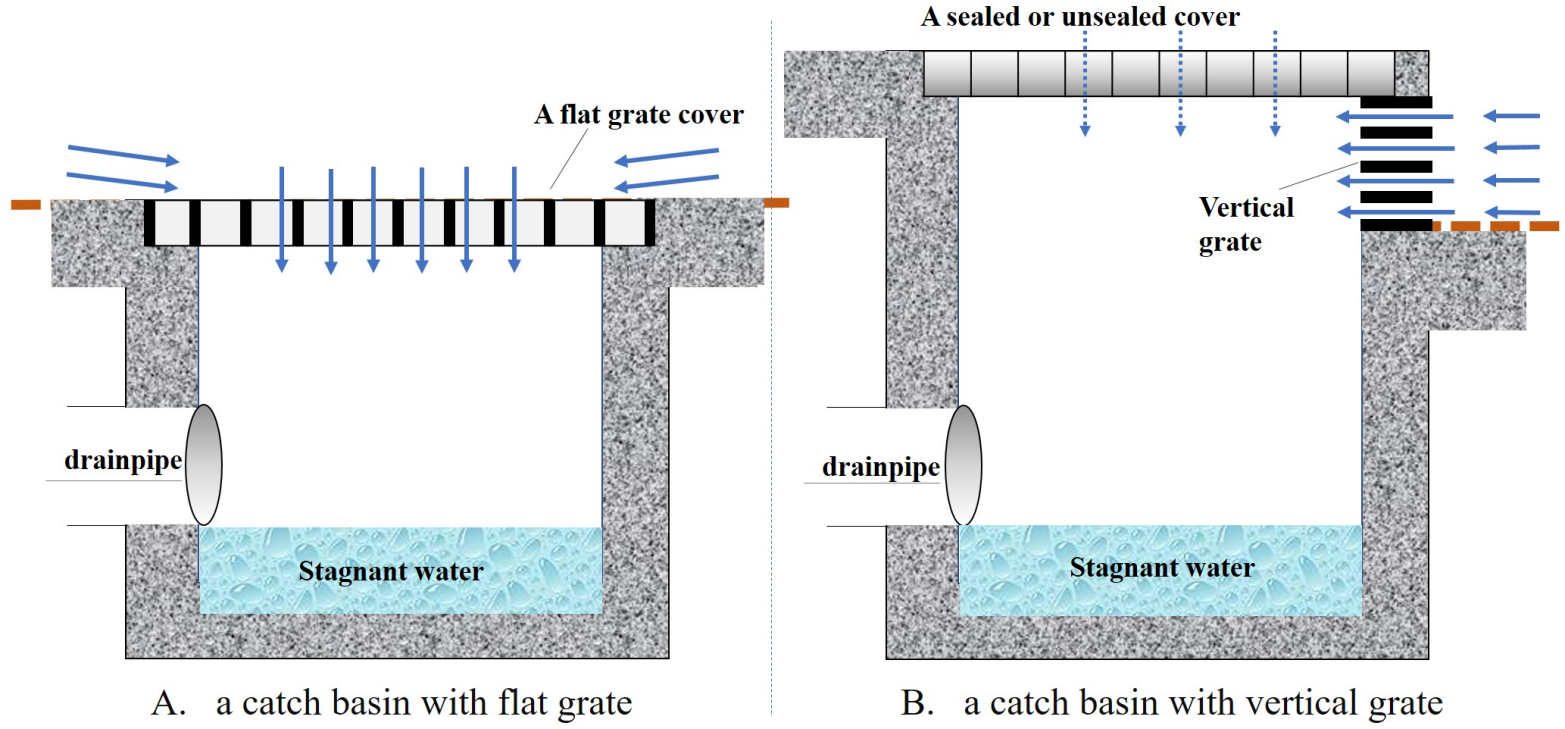
A cross-sectional diagram of CBs with a flat grate (A) and a vertical grate (B).

**Fig 3:**
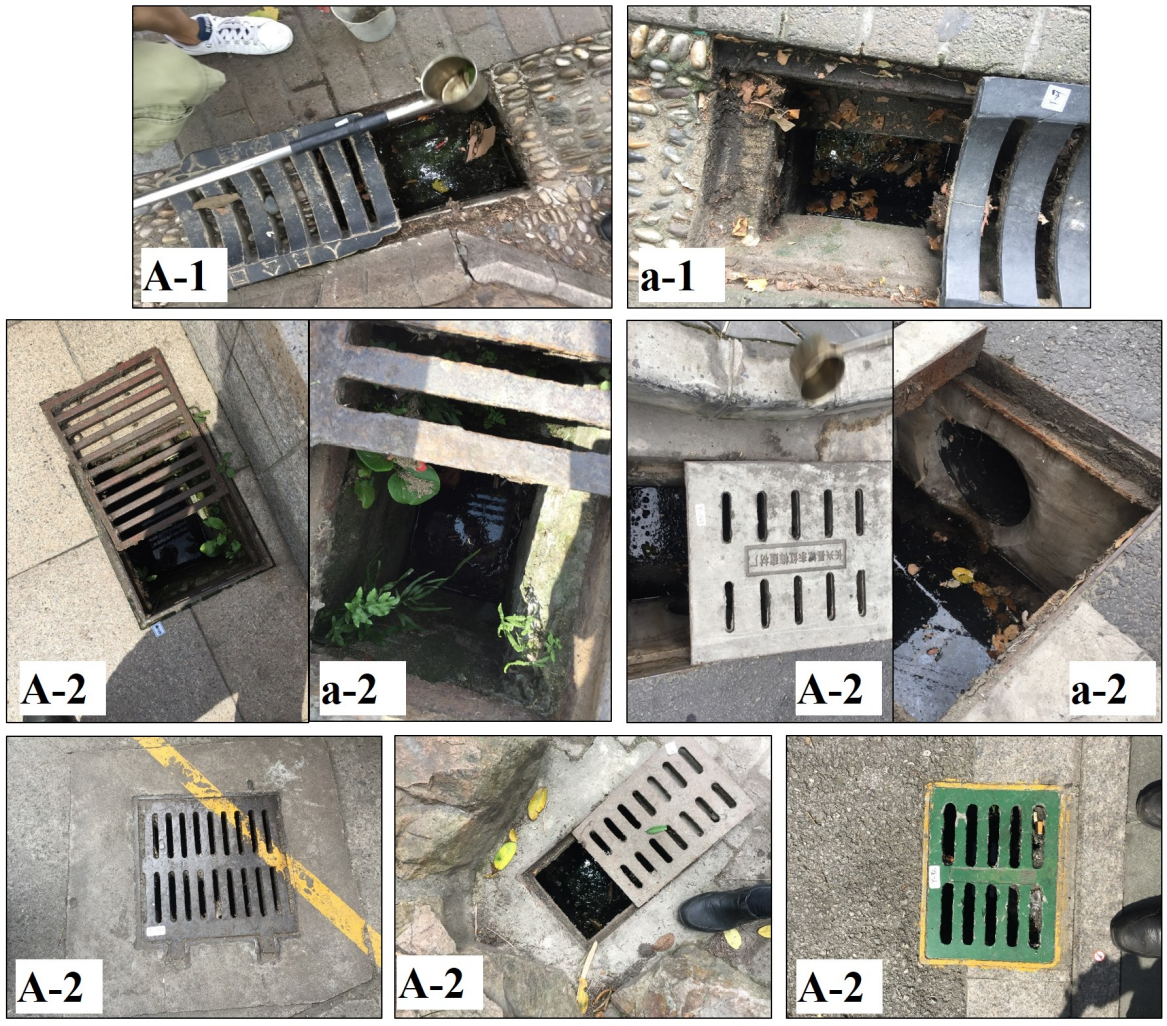
**Examples of CBs with flat grates**, including those (A-1) connected with surface gutters, and those (A-2) that are independent; (a-1,2) the insides of CB chambers.

**Fig 4:**
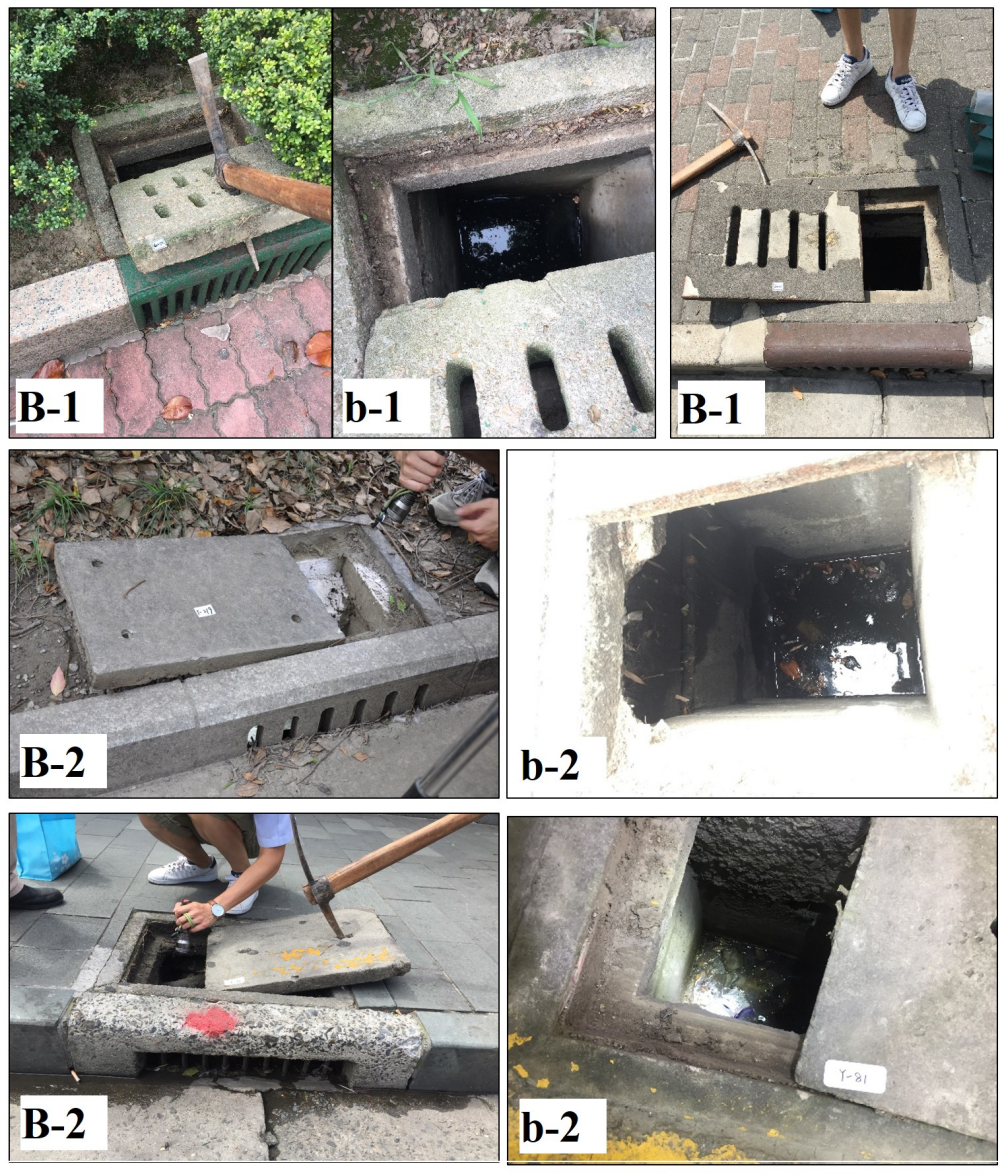
**Examples of CBs with vertical grates**: including those (B-1) with unsealed lids, and those (B-2) with sealed lids; (b-1,2) the insides of CB chambers.

The CBs surveyed in this study were those with flat or vertical grates (Figs 2–4). These forms are designed to allow urban surface runoff to flow directly into the chambers, and then further drain off through underground pipelines. Structurally, there is a space between the bottom of the chamber and the level of the outflow pipe, which often allows stagnant water to pool for a period long enough to allow mosquitoes to breed. Urban runoff is mainly from precipitation, street washing, car washing, lawn irrigation and other sources.

There are many types of CBs in downtown Shanghai. The CBs with flat grates include those connected with surface gutters and other that are independent (Fig 3). The below-ground parts of the former type tend to have two connecting drainpipes. The CBs with a vertical grate are more common on streets or in areas with roadways. These can be further categorized into two subtypes according to whether the lids are sealed or unsealed (Fig 4).

#### Manhole chambers (MCs)

**Fig 5:**
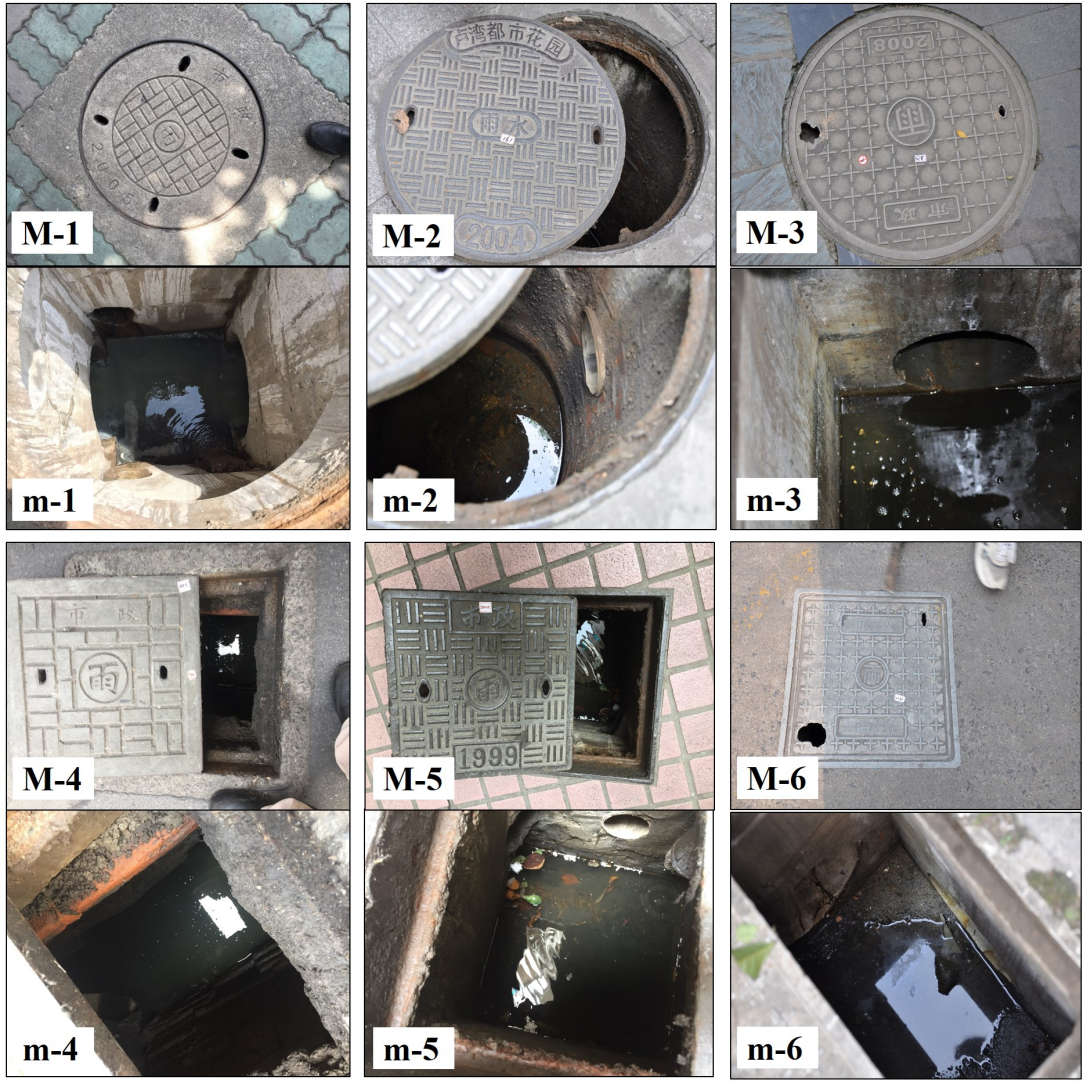
Examples of stormwater MCs with different sizes and covers, including those (M-1,2,3) with round covers, and those (M-4,5,6) with square cover, and covers made of (M-1,4) concrete, (M-2,5) cast iron, and (M-3,6) plastic composite. (m-1,2,3,4,5,6) the insides of MCs.

**Fig 6:**
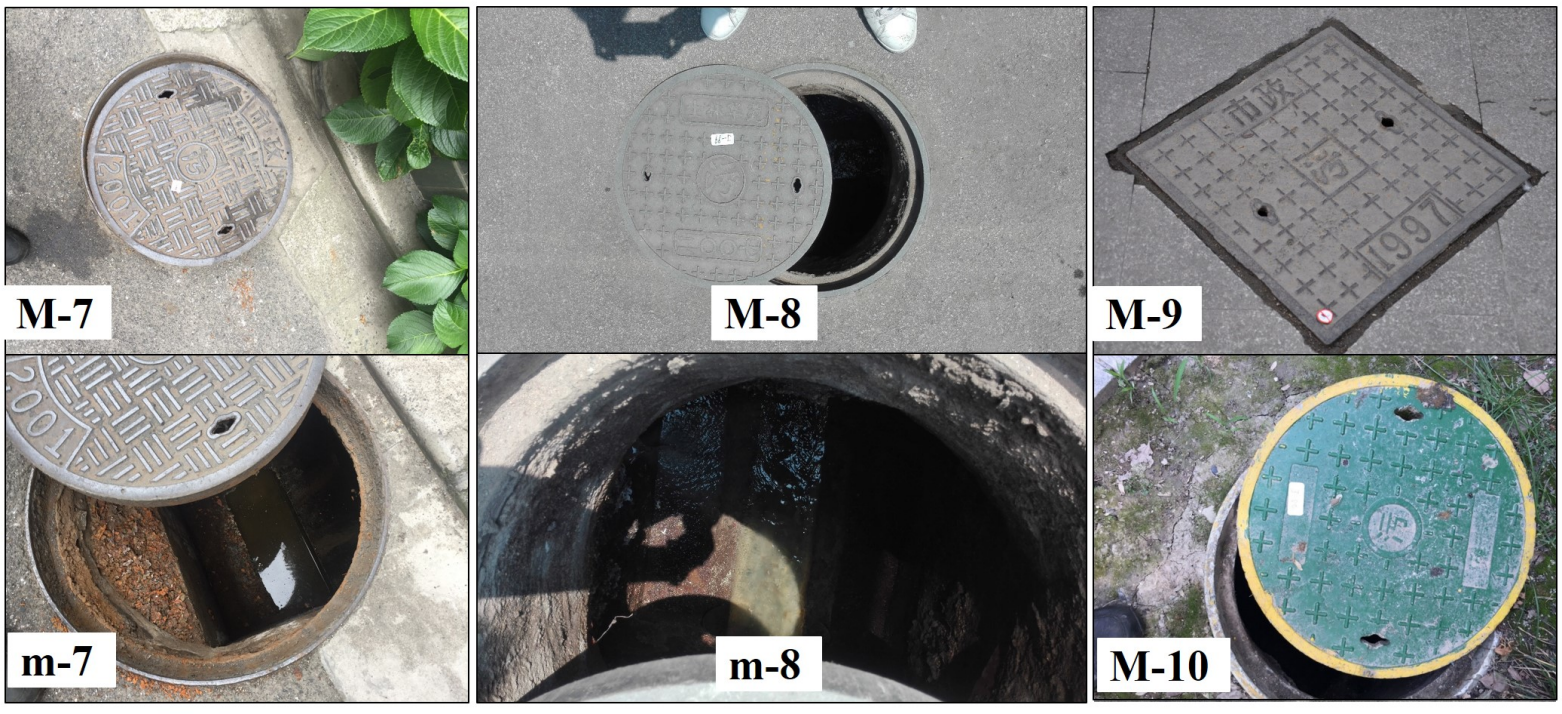
Examples of sewage MCs with different sizes and covers. Sewage MCs include those covers made of (M-7,9) cast iron, and (M-8,10) plastic composite. (m-7,8) the insides of MCs.

**Fig 7:**
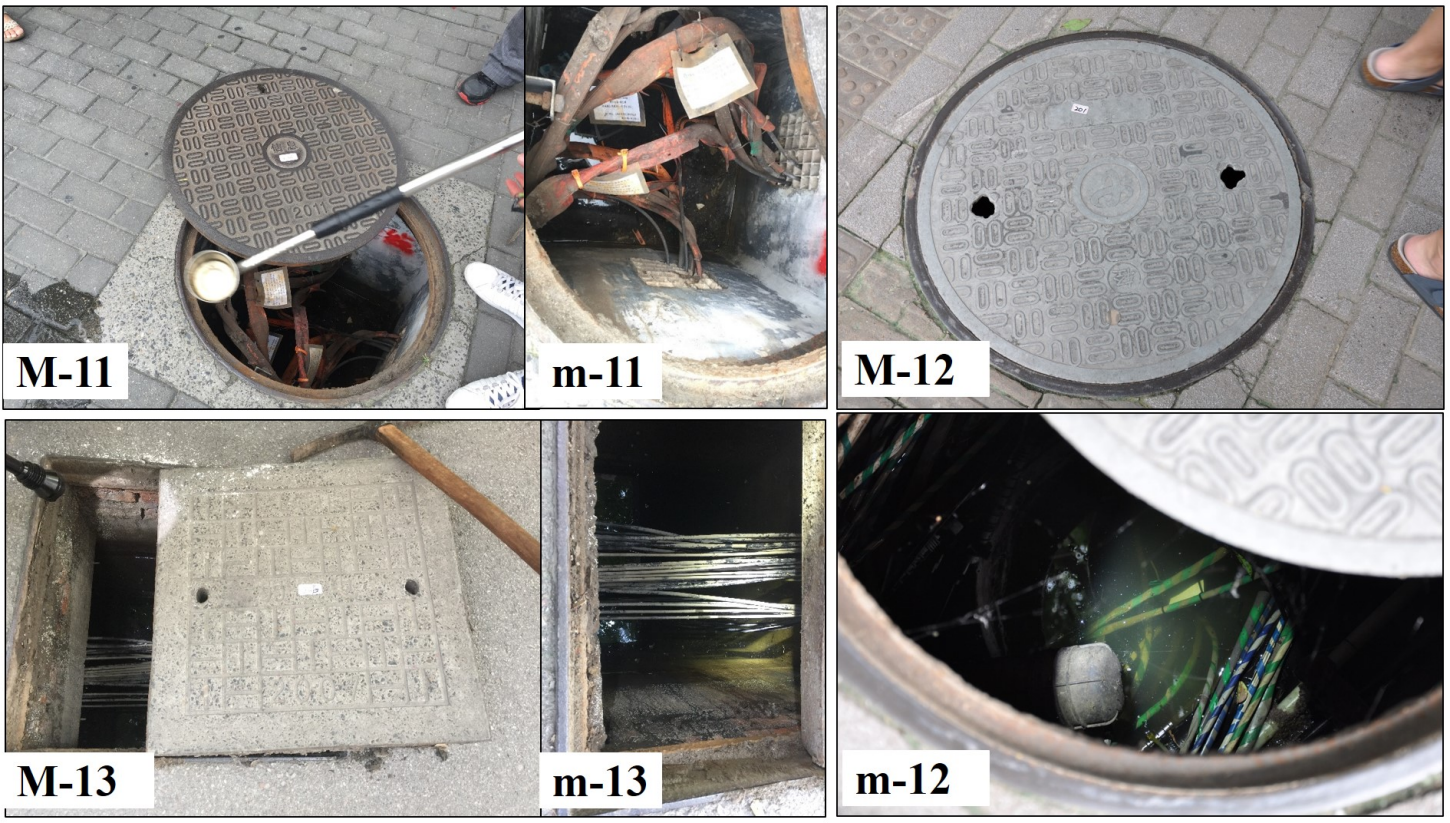
Examples of telecommunication MCs with different sizes and covers. Telecommunication MCs include those covers made of (M-11) cast iron, (M-12) concretes, and (M-13) plastic composite. (m-11,12,13) the insides of MCs.

The MCs surveyed in this study were structures with large covers and large below-ground chambers. The stagnant or slow-moving water inside the MC chambers was not directly from surface runoff; for this reason, the covers were usually not designed as grated types. Instead, the MC covers were more often designed with two or four small pick holes for lifting. There is often more than one pipe inside the chamber. The MCs in downtown Shanghai include diverse types with different structural features or designs serving different purposes. The most common types are MCs with covers engraved with word “雨” in Chinese (Fig 5); these are used for stormwater drainage. There are also manholes with covers engraved with words “污” (Fig 6) or “信息” in Chinese (Fig 7); these are used for domestic sewage discharge and telecommunication, respectively.

### Sample size and characteristics of drainage systems

A total of 858 CBs or MCs were examined. Each structure was numbered, and photos were taken on the spot. The lids were lifted to measure the chamber size and the water-holding capacity. The following data were recorded in the field for each structure: 1) location: community and environmental types; 2) structural types: CB or MC and its function (stormwater, sewage, telecommunications, or other usage); 3) cover size: classified as large (diameter or width ≥0.6m) or small/medium (diameter or width < 0.6m); 4) features or designs: type of grate, type of cover material and openness; 5) the depth of the inside chamber (m); 6) presence or absence of water. For structures containing water, we also recorded 7) the water-holding status (stagnant or running water); 8) water depth(cm); 9) temperature of the water (°C); 10) presence or absence of larvae; if there were larvae in the water, then we recorded 11) larval density (no. of larvae per dip).

### Sampling of immature mosquitoes

For structures with larvae present, sampling was conducted and larval density was measured. A dipper (500ml) with a long handle (1.5m) was used to sample for mosquito larvae. Each structure was sampled for larvae by quickly sweeping the dipper along the water surface of the structure’s corner, which is the larvae’s preferred habitat, and then counting and recording the number of larvae per dip. Sampled larvae in the dipper were transferred to a plastic mineral water bottle (600ml) marked with the sample information (sample number, location, and date), and the process was repeated twice for each structure following short waiting periods to allow the larvae to resurface. All larvae collected were returned to the medical entomology laboratory of the local CDC, then reared in different cages according to sampling locations. The artificial rearing maintenance last 20 days, after which the emerged adult mosquitoes were counted and identified.

### Statistical analysis

Data were analyzed using the SPSS version 11.5 (SPSS, Inc., Chicago, IL, U.S.A.) statistical package. Differences between percentages were compared using Pearson Chi-square test. Most of quantitative data were not normally distributed. After logarithmic transformation, independent t-test or one-way ANOVA (analysis of variation) were applied for analysis. A significance level of P<0.05 represented a significant difference.

## Result

### Summary of mosquito collection

A total of 539 stormwater CBs and 309 MCs were examined for mosquito production in the cross-sectional study. The study area included 12 green lands, 23 residential neighborhoods and 15 roadways within 10 communities (Fig 1). No significant differences in water-holding percentage were found among the above three environment types for both CBs and MCs (Table 1).

**Table 1:** Percentage of CBs and MCs containing water in different environments.

Environmental types	CBs		MCs	
	n1 / n0	Water-holding percentage / %	n2/n0	Water-holding percentage / %
Green lands	80 / 163	49.08	47 / 64	73.44^#^
Neighborhoods	135 / 269	50.19	144 / 175	82.29^#^
Streets or Roadways	57 / 147	38.78	56 / 70	80.00^#^
Sum	272 / 549	49.54	247 / 309	79.94^#^

n1: No. of CBs containing water; n2: No. of MCs containing water; n0: No. of CBs or MCs surveyed

^#^ Pearson X^2^ test, compared with CBs, water-holding percentage is significantly greater, p value < 0.05.

**Fig 8:**
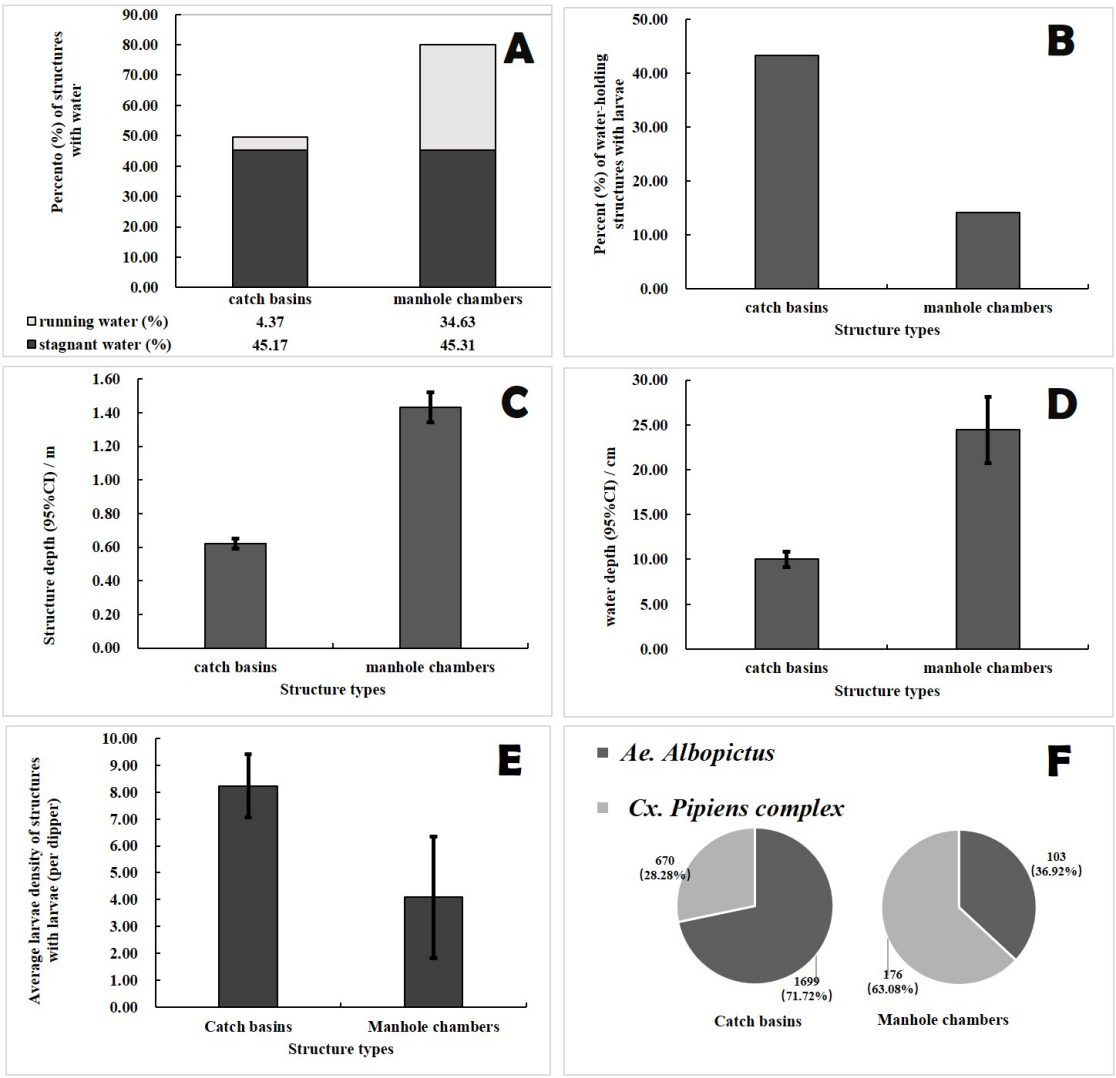
Comparison of structural characteristics and mosquito productions between CBs and MCs.

Overall, 79.9% of MCs examined were found with standing water, which was significantly higher than the 47.0% of CBs (X^2^ = 76.407, P < 0.001). However, the CBs were characterized by a greater percentage of stagnant water (CBs 45.2% vs. MCs 35.3%, X^2^ = 11.465, P = 0.001) due to the higher percentage of MCs with running/flowing water (MCs 34.6% vs. CBs 4.4%, X^2^ = 223.928, P < 0.001) (Fig 8A). In terms of structural and physical properties, MCs in downtown Shanghai were characterized by both greater chamber depth and water depth than CBs (chamber depth: 1.43 vs. 0.62m, t = -16.712, P < 0.001; water depth: 24.43 vs. 10.03 cm, t = -7.425, P < 0.001) (Fig 8C and 8D).

There were exclusively two species of larvae found in the drainage systems, *Ae*. *albopictus* and *Cx*. *pipiens* complex. Female mosquito apparently preferred to lay eggs in CBs more than in MCs, since we found more water samples with larvae present and higher larval density in water-holding CBs than in MCs (percentage with larvae: CBs 43.4% vs. MCs 14.2%, X^2^ = 53.136, P < 0.001; larval density: CBs 8.23 vs. MCs 4.09 per dip, t = 3.287, P = 0.001) (Fig 8B and 8E). Moreover, the predominant species found in CBs was *Ae*. *albopictus* (constituent ratio: 71.7% of *Ae*. *albopictus* vs. 28.3% of *Cx*. *pipiens*, X^2^ = 893.914, P < 0.001), which was just the opposite for MCs (constituent ratio: 36.9% of *Ae*. *albopictus* vs. 63.1% of *Cx*. *pipiens*, X^2^ = 38.201, P < 0.001) (Fig 8F).

**Fig 9:**
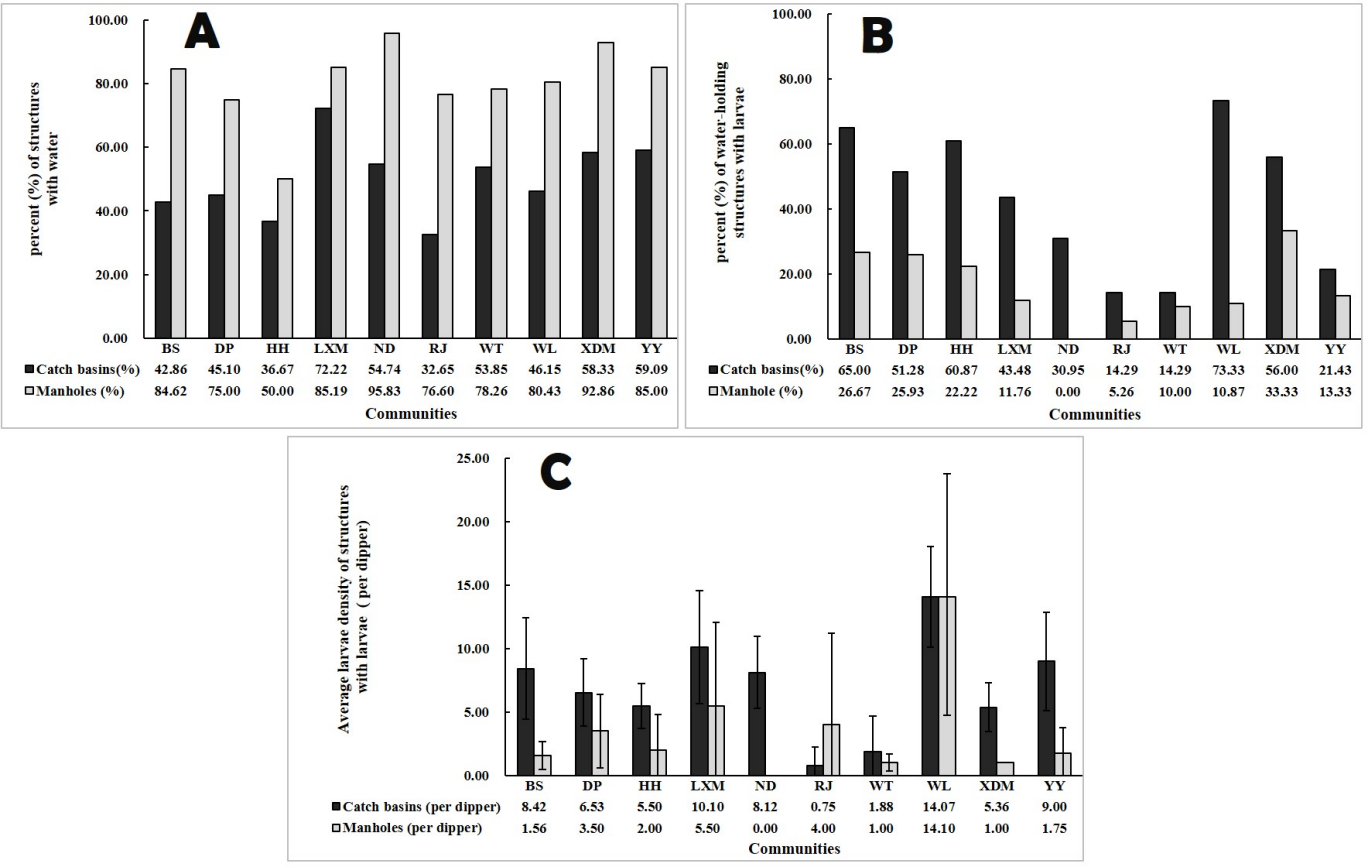
Water-holding status and mosquito productions of CBs and MCs among 10 communities.

As shown in Fig 9, all parameters of CBs and MCs varied widely among the 10 communities. For CBs, the percentage with standing water ranged from 32.7% to 72.2%, the larvae-positive percentage of water samples ranged from 14.3% to 73.3%, and larval density ranged from 0.75 to 14.07 per dipper. This variation also held true in MCs (Fig 9).

### Characteristics of catch basins and mosquito production

As mentioned above, CBs in downtown Shanghai can be sorted into those with flat grates and those with vertical grates. Because of the structural difference, the latter type are deeper in both chamber depth and water depth than the former type (chamber depth: 0.76 vs. 0.56 m, t = 6.525, P < 0.001; water depth: 11.43 vs. 9.45 cm, t = 2.090, P = 0.038). Moreover, the vertical grate form yielded higher breeding percentage than the flat grate type (percent with larvae: 51.3% vs. 40.1%, X^2^ = 2.856, P = 0.091) while no difference in larval density was realized (8.78 vs 7.94, t = 0.672, P = 0.503). CBs with a vertical grate and an unsealed lid were the structures most conducive to larval production with 60.0% of water-holding ones containing larvae and a larval density of 12.36 per dip; flat grates connected with gutters were the least conducive to larval production with 30.6% of water-holding ones containing larvae and a larval density of 3.23 per dip (Table 2).

**Table 2:** Physical parameters and mosquito production of CBs with different features.

Category of CBs		No. of CBs containing water	No. (%) of water-holding CBs with larvae	Mean larval density (95% CI)	larvae range per dip (min~max)	depth of structure / m	depth of water / cm	No. (%) with running water
CBs with								
	Flat grates: a. connected with gutters	36	11 (30.6)	3.23 (1.98, 4.47)	0~10	0.42	10.83	0 (0.0)
	Flat grates: b. independent	156	66 (42.3)	8.72 (7.44, 19.20)	0~60	0.78	9.13	17 (10.9)
	Flat grates: Sum	192	77 (40.1)	7.94 (6.46, 9.41)	0~60	0.56	9.45	17 (8.9)
	Vertical grates: a. sealed cover	50	23 (46.0)	5.98 (3.93, 8.02)	0~30	0.83	12.21	7 (14.0)
	Vertical grates: b. unsealed cover	30	18 (60.0)	12.36 (8.87, 15.85)	0~40	0.65	10.13	0 (0.0)
	Vertical grates: Sum	80	41 (51.3)	8.78 (6.79, 10.77)	0~40	0.76	11.43	7 (8.8)
CBs located in								
	Green lands	80	26 (32.5)	6.25 (4.62, 7.88)	0~20	0.65	11.56	1 (1.3)
	Residential neighborhood	135	71 (52.6)	10.12 (8.36, 11.88)	0~60	0.53	8.14	12 (8.9)
	Streets or roadways	57	21 (36.8)	4.29 (2.84, 5.73)	0~20	0.79	12.34	11 (19.3)
Sum		272	118 (43.4)	8.23 (7.05, 9.41)	0~60	0.62	10.03	24 (8.8)

*Aedes albopictus* was found in 93.5% of larvae-positive CBs, 29.0% of which also contained *Cx*. *pipiens* complex. Water samples with *Ae*. *albopictus* present were almost equal between CBs with flat and vertical grates (94.1% vs. 93.2%, respectively; X^2^ = 0.035, P = 0.851), while *Ae*. *albopictus* constituent ratio of the vertical type was a little higher than that of the flat type (74.2% vs. 68.3%, X^2^ = 9.721, P = 0.002) (Table 3).

**Table 3:** Mosquito species composition of CBs with different features.

		No. of sampling CBs	*Ae*. *Albopictus*			*Cx*. *pipiens* complex			No. (%) of CBs with both *aedes* and *culex*
			No. (%) of CBs with *Ae*. *albopictus*	count	species constituent ratio / %	No. (%) of CBs with *Cx*. *Pipiens*	count	species constituent ratio / %	
CBs with									
	Flat grates: Sum	34	32 (94.1)	682	68.3	12 (35.3)	316	31.66	10 (29.4)
	Vertical grates: Sum	73	68 (93.2)	1017	74.2	26 (35.6)	354	25.82	21 (28.8)
CBs located in									
	Green lands	25	20 (80.0)	382	53.7	17 (68.0)	329	46.27	12 (48.0)
	Residential neighborhood	71	70 (98.6)	1230	82.9	17 (23.9)	254	17.12	16 (22.5)
	Streets or roadways	11	10 (90.9)	87	50.0	4 (36.4)	87	50.00	3 (27.3)
Sum		107	100 (93.5)	1699	71.7	38 (35.5)	670	28.28	31 (29.0)

The CBs located in residential neighborhoods were the most conducive to larval production; 52.6% contained standing water, and larval density was 10.12 per dip. These values were much higher than the corresponding measures for CBs located in green lands and roadways (larvae-positive percentage: respective Pearson Chi-square values: X^2^ = 8.190 and 3.984, P = 0.02 and 0.04; larval density by one-way ANOVA, F = 8.583, P < 0.001). Moreover, CBs in residential neighborhoods yielded more *Ae*. *albopictus* characterized by a higher *albopictus*-positive percentage (98.6%) and a higher *albopictus* constituent ratio (82.9%)(Table 3).

### Characteristics of manhole chambers and mosquito production

Based on usage, MCs were classified into three major varieties: MCs of stormwater, sewage and telecommunications; each type had its own special features (Figs 5–7). MCs of sewage had the highest percentage of running water (57.6%) and the lowest larval presence (only 1 out of 33 observations, 3.0%). However, the single larvae-positive case produced the highest larval density among all MCs examined in this study. MCs used in telecommunications featured the lowest percentage of running water as well as the deepest chamber depth (mean 2.11 m) and water depth (mean 65.65 cm). An interesting observation was that the above two MCs types shared a common feature that *Ae*. *albopictus* was present in most of these MCs (100.0% and 83.3%, respectively) but their constituent ratios of *Ae*. *albopictus* were very low (only 7.6% and 11.4%, respectively) (Table 4).

**Table 4:** Physical parameters and mosquito production of MCs with different features.

Category of MCs		No. of MCs containing water	No. (%) of water-holding MCs with larvae	Mean larval density (95% CI)	larvae range per dipper (min~max)	depth of structure / m	depth of water / cm	No. (%) with running water
MCs for								
	Stormwater (SMCs)	168	28 (16.7)	2.70 (1.73, 3.66)	0~15	1.27	16.04	85 (50.6)
	Sewage	33	1 (3.0)	55.00 (-8.53, 118.53)	0~60	1.24	9.67	19 (57.6)
	Telecommunication	46	6 (13.0)	2.08 (0.32, 3.85)	0~10	2.11	65.65	3 (6.5)
Sum (total manholes)		247	35 (14.2)	4.09 (1.83, 6.34)	0~60	1.43	24.43	107 (43.3)
MCs for stormwater (SMCs) with different covers								
	Size 1: large	28	3 (10.7)	0.83 (0.40, 1.26)	0~1	1.93	27.86	17 (60.7)
	Size 2: moderate or small	140	25 (17.9)	2.92 (1.85, 3.99)	0~15	1.14	13.68	68 (48.6)
	shape1: square	125	22 (17.6)	3.07 (1.86, 4.28)	0~15	1.11	14.10	61 (48.8)
	shape2: round	43	6 (14.0)	1.33 (0.84, 1.83)	0~3	1.32	16.32	24 (55.8)
	material 1: concrete	66	4 (6.1)	1.00 (0.23, 1.77)	0~3	1.29	16.73	49 (74.2)
	material 2: cast iron	67	13 (19.4)	2.88 (1.29, 4.48)	0~15	1.31	16.91	26 (38.8)
	material 3: plastic composite	35	11 (31.4)	3.09 (1.43, 4.75)	0~10	1.17	13.09	10 (28.6)
MCs for stormwater (SMCs) located in								
	Green lands	41	3 (7.3)	0.83 (0.40, 1.26)	0~1	1.52	23.34	16 (39.0)
	Residential neighbourhood	106	23 (21.7)	3.09 (1.94, 4.23)	0~15	1.21	14.58	52 (49.1)
	Streets or roadways	21	2 (9.5)	1.00 (-1.25, 3.25)	0~3	1.10	9.19	17 (81.0)

The most ubiquitous MCs were those used for stormwater (abbreviated as SMCs). Of these, 16.7% were larvae-positive, with an *Ae*. *albopictus* constituent ratio much higher than in the other two MCs categories (55.0% vs. 7.6%, 11.4%, X^2^ = 43.964 and 26.797, P < 0.001 for both tests). The SMCs also came in a variety of shapes and sizes, ones with covers of small/medium size, square-shaped, and made of plastic composite were more conducive to mosquito production, with mean larval densities of 2.92, 3.07 and 3.09 per dip, respectively. Larval presence in the plastic type was 31.4%, much higher than in the other designs (P <0.01 for both comparisons; Pearson Chi-square tests). The SMCs with cast iron covers yielded the most *Ae*. *albopictus*, as 88.9% were found with *Ae*. *albopictus*, and the species constituent ratio was 86.2%, which was much higher than in the SMCs with concrete or plastic lids (86.2% vs. 31.3%, 41.8%, X^2^ = 19.848 and 29.512, P < 0.001 for both comparisons) (Table 5).

**Table 5:** Mosquito species composition of MCs with different features.

		No. of sampling MCs	*Ae*. *Albopictus*			*Cx*. *pipiens* complex			No. (%) of MCs with both *aedes* and *culex*
			No. (%) of MCs with *Ae*. *albopictus*	count	species constituent ratio / %	No. (%) of MCs with *Cx*. *pipiens*	count	species constituent ratio / %	
MCs for									
	Stormwater (SMCs)	28	22 (78.6)	93	55.0	9 (32.1)	76	45.0	3 (10.7)
	Sewage	1	1 (100.0)	5	7.6	1 (100.0)	61	92.4	1 (100.0)
	Telecommunication	6	5 (83.3)	5	11.4	4 (66.7)	39	88.6	3 (50.0)
Sum (total manholes)		35	28 (80.0)	103	36.9	14 (40.0)	176	63.01	7 (20.0)
MCs for stormwater (SMCs) with different covers									
	Size 1: large	3	1 (33.3)	1	10.0	2 (66.7)	9	90.0	0 (0.0)
	Size 2: moderate or small	19	16 (84.2)	95	58.6	7 (36.8)	67	41.4	4 (21.1)
	shape1: square	16	13 (81.3)	84	55.6	7 (43.8)	67	44.4	4 (25.0)
	shape2: round	6	4 (66.7)	12	57.1	2 (33.3)	9	42.9	0 (0.0)
	material 1: concrete	4	2 (50.0)	5	31.3	3 (75.0)	11	68.8	1 (25.0)
	material 2: cast iron	9	8 (88.9)	50	86.2	2 (22.2)	8	13.8	1 (11.1)
	material 3: plastic composit	9	7 (77.8)	41	41.8	4 (44.4)	57	58.2	2 (22.2)
MCs for stormwater (SMCs) located in									
	Green lands	3	1 (33.3)	1	10.0	2 (66.7)	9	90.0	0 (0.0)
	Residential neighbourhood	17	14 (82.4)	89	57.8	6 (35.3)	65	42.2	3 (17.7)
	Streets or roadways	2	2 (100.0)	6	75.0	1 (50.0)	2	25.0	1 (50.0)

The SMCs located in residential neighborhoods produced more larvae than those in green lands and roadways (larvae present: 21.7% vs. 7.3%, 9.5%, respectively; X^2^ = 4.200 and 1.643, P = 0.040 and 0.200; larval density: 3.09 vs. 0.83, 1.00 per dip, respectively; no statistical test was conducted due to the small sample size), and 14 sampling SMCs of residential neighborhoods out of 17 were found with *Ae*. *albopictus* (82.4%) (Table 5).

## Discussion

To our best knowledge, This is the first detailed report on mosquito production from the near-surface urban drainage system in Shanghai, China. The system contains hundreds of thousands of CBs and MCs with a variety of designs or features. Considering the poor accessibility, irregular timing of flooding, and fluctuation of water volume, this vast system is a challenge for urban mosquito control. However, this study may provide some constructive suggestions for mosquito control in urban drainage systems since we explored the association between larval abundance and features or locations of the drainage devices, and indicated the types or designs of these structure which were more conducive to *Ae*. *albopictus* production in downtown Shanghai.

Stormwater infrastructure has been identified as a major source of mosquitoes in many metropolitan areas [19], especially in CBs. Rydzanicz concluded that CBs served as major development and resting sites for anthropophilic and zoophilic mosquitoes in urban Poland [15], Harbison suggested that CBs (numbers up to 200,000) are the primary source of potentially disease-carrying mosquitoes in the Chicago metropolitan area [19]. Kobayashi confirmed that catch basins were the main larval habitats in urban environments of Japan[20], and herein we have shown that this also holds true in urban Shanghai. Our study demonstrated that nearly half (49.5%) of CBs examined held water and that a fairly high percentage (43.4%) contained larvae, suggesting that the actual number could be much higher than the conservative estimates 10% by Su [9]. In urban Shanghai, there are countless CBs distributed in numerous green lands, residential neighborhoods, business centers and streets or roadways. The fact that 21.5% (118 out of 549) CBs harbored mosquito larvae means more than one in five of these structures may continuously produce mosquitoes. If correct, there would be no doubt that CBs constitute a major mosquito-producing sources in the city.

We found that CBs were more conducive to mosquito production than MCs, which may be due to physicochemical characteristics of water and the structurally open nature of CBs. Water accumulation in CBs results mainly from stormwater and dry weather urban runoff, which picks up and carries a variety of suspended, dissolved, and floating pollutants into the CBs chamber [19], forming aquatic habitats rich in organic matter and debris that are highly favorable to mosquito production. Compared with MC covers having only 2~4 small pick holes, the flat or vertical grates of CBs provide ideal pathways for mosquitoes flying in or out; this, coupled with micro-environments that lack natural enemies (predator-free zones) and difficult accessibility, allow uncontrolled mosquito breeding in CBs.

Numerous studies have suggested that organically-rich (polluted) stormwater runoff is more conducive to breeding by *Culex* mosquitoes, especially for vectors of West Nile virus [19]. In southern California, *Cx*. *quinquefasciatus* is the predominant or sole species found in belowground stormwater infrastructures including CBs [9, 21–25]. Studies form cities in Florida and Mexico also found that *Cx*. *quinquefasciatus* was the most common species sampled from stormwater drains and CBs [11, 13]. Jackson in Canada suggested *Cx*. *pipiens* was much more abundant in CBs than in other water bodies[26], and Rydzanicz in Poland found *Cx*. *pipiens* s.l. and *Cx*. *torrentium* was the most prevalent species in all CBs as the predominant species[15]. In China, an earlier study in Dalian located in northern China also suggested that *Cx*. *pipiens pallens* was the only species found in urban CBs [27]. However, we obtained different results for the mosquito species composition in CBs in downtown Shanghai. The fairly large sample size in this study showed that 71.7% of larvae sampled from CBs belonged to *Ae*. *albopictus*, and *Ae*. *albopictus* was present in 93.5% of larvae-positive CBs. This difference may be attributed to several causes. First, in urban Shanghai *Ae*. *albopictus* was predominated, especially in residential neighborhoods [28]; a large population of female *Ae albopictus* need to find nearby aquatic habitats for oviposition due to the species’ limited flying distance [29]; this is in contrast to regions of Europe or America, where *Ae*. *albopictus*’ lesser abundance is partly attributed to the relatively short duration after local introduction [30–32]. For examples, *Ae*. *albopictus* was collected occasionally in CB samples in Florida, with quite small prevalence (0.06%). Secondly, due to degradation of natural habitats and outstanding adaptability, *Ae*. *albopictus* can colonize new ecological niches [15], developing previously unknown oviposition preference for CBs; third, the water bodies in CBs are relatively small in size and contain more stagnant water compared with MCs, and plant debris or floating trash in the water provide ideal substrates for *Aedes* oviposition. Studies in Japan have shown that the larvae of *Ae*. *albopictus* breed in catch basins along public roads and by public facilities and detached houses (detailed data unpublished). To our knowledge, this is the first record of *Ae*. *albopictus* as the predominant species in stormwater infrastructure.

**Fig 10:**
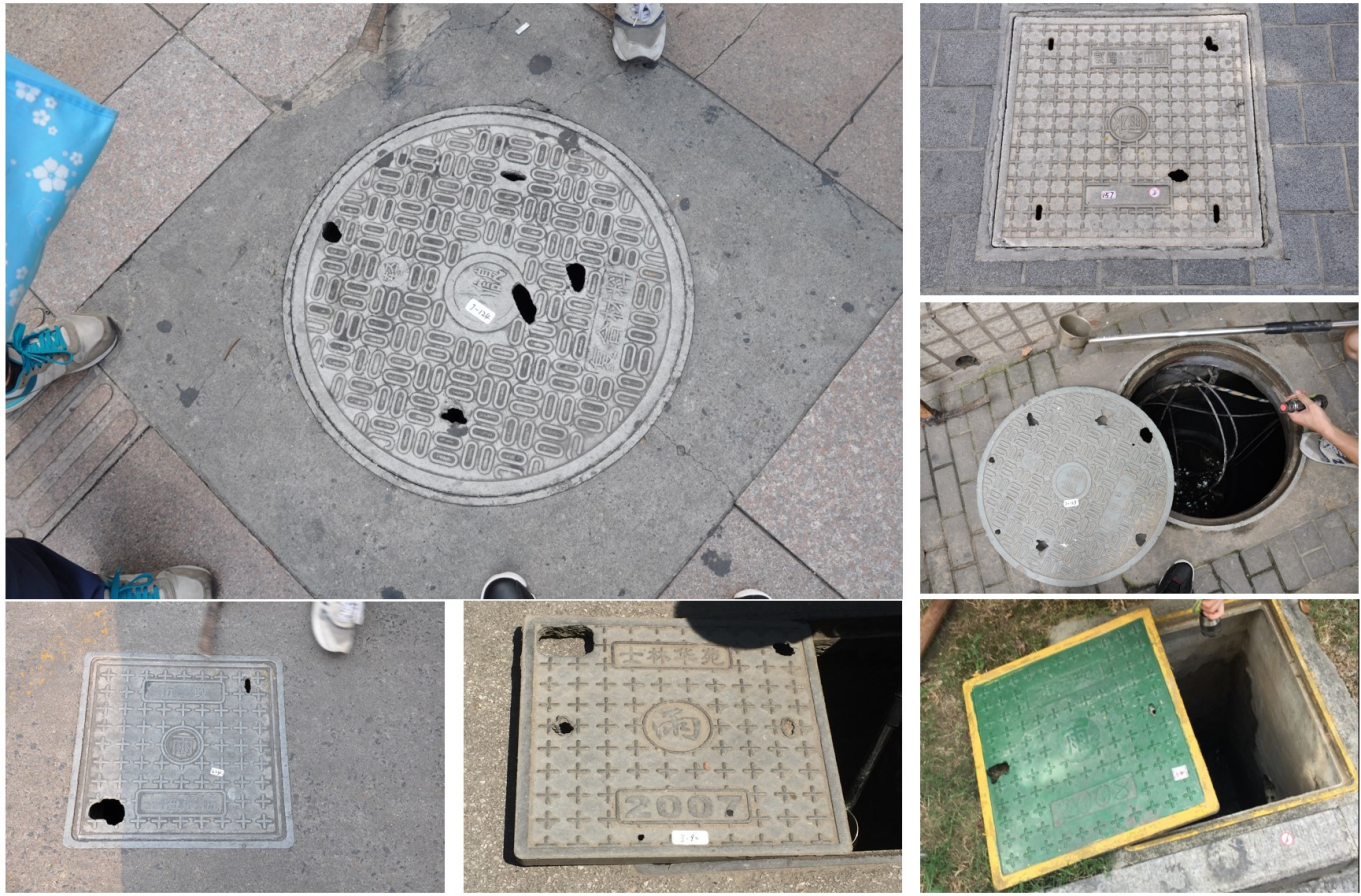
Examples of aging and broken MCs covers made of plastic composite.

Our study suggested that most mosquito females prefer to oviposit in stagnant water, meaning that a considerable portion of MCs with running water (34.6%) did not support mosquito production. This along with the relatively limited access for the mosquitoes meant that significantly fewer mosquitoes were found in MCs of downtown Shanghai. In this study, few of the sewage MCs examined were larvae-positive, and this was probably because the running (57.6%) and over-polluted water was not favorable for mosquito production. The only MC with larvae present may have been misclassified by its cover, which were marked with the word “污”, while its inside chamber was more like that of a stormwater type. The MCs in urban Shanghai featured multifarious designs, especially in the chamber cover, which are physically characterized by different sizes, shapes and materials. However, all MCs shared one common feature in that two or four pick holes could always be found on the covers. As suggested by Metzger [5], adult female mosquitoes may penetrate openings as small as 1/16 inch (2mm) to gain access to water for egg laying; the cover pick holes observed in this study were much larger than that particular threshold, meaning that female mosquitoes could enter and oviposit if the water was favorable. Another important finding was that covers made of plastic composite were vulnerable to physical aging and becoming fragile, so that more broken MCs were found among those with plastic covers (Fig 10). This allowed more mosquito females to enter and deposit eggs inside, which could explain why MCs with plastic covers were always characterized by a higher percentage of larval presence (31.4%).

It should be noted that *Ae*. *albopictus* was also found in most water samples (80.0%) of MCs with larvae, which further demonstrated that *Ae*. *albopictus* can tolerate quite a wide range of aquatic habitats, including relatively large water bodies such as those inside the MCs in downtown Shanghai. This is especially true for stormwater MCs, whose *Aedes* constituent ratio was greater than that of *Culex*. Although most of the samples showed low larval densities ranging from 0 to 15 larvae per dip, considering the tremendous baseline amount, these low larval densities in total aggregate constitute another major mosquito sources besides CBs in urban Shanghai.

Location can greatly affect whether a drainage structure becomes a significant source of mosquitoes [5]. Among the 10 communities and three sampling environment types, identical structures in different locations varied widely in potential mosquito production, which may be attributed to a variety of factors including environmental, human behavioral, construction and local factors operating singly or in combination [5]. First, water-holding status mainly determined by the amount of rainfalls, and dry season runoff could be an essential factor affecting mosquito development and production. The water-holding status could vary greatly depending on runoff quantity, quality and frequency of events like car washing, street washing or lawn irrigation. Impermeable cement ground surface produces more runoff with organic matter than green lands, since the latter can capture and detain more water; however, green lands may provide more plant debris flowing into the drainage system, which could alter aquatic status for mosquito habitats. Second, adult mosquito species composition and density could also affect larval production in local drainage structures; the adult population is mainly determined by surrounding refuges and potential host available. In Shanghai, there are more *Ae*. *albopictus* in residential neighborhoods than in green lands, since the former provide more human hosts for the anthropophilic tiger mosquito to feed on [28]. This may explain why CBs and MCs located in residential neighborhoods produce more *Ae*. *albopictus* larvae.

The predominance of *Ae*. *albopictus* breeding in residential neighborhoods dense in human residents would produce more concerns for public health, since a large population of *Aedes* vectors companied by a large population of human hosts would greatly facilitate disease transmission if *Aedes*-borne pathogens are present. *Aedes albopictus* has been the most dangerous vector species in Shanghai, especially after the first local dengue infection was reported in 2017 [33]. We suggest that *Aedes* control in Shanghai should focus on CBs and other potential larval habitats in and around residential neighborhoods.

If it is not feasible to add sealed lid covers to the CBs to prevent entry of adult mosquitoes, then we can only find solutions dealing with water or water-holding status in addition to larvicide application. We found that some catch basins under poor maintenance were nearly filled to the outflow pipe level with soil or debris, and thus could not retain water or support mosquito production. This model bears some resemblance to the design proposed by Rydzanicz, who suggested that catch basins should be redesigned by lowering the inlet/outlet pipe location to prevent water or debris accumulation and thus mosquito larvae development [15]. We are cautiously optimistic concerning this model since the space between the chamber bottom and the outflow pipe was used to retain sediments such as sand and other debris from entering and blocking the outgoing drainage system. This model can only be applied in areas with good sanitary condition where little sand or debris are washed into the basin. We recommended permeable construction materials being used for ground surface and catch basin chamber to allow excessive runoff to replenish underground aquifers and decrease downstream erosion and pollution. In addition, water accumulation in the basins could permeate into soil without forming stagnant water. For the already existing CBs with stagnant water, copepods as effective mosquito predators, particularly in artificial containers, can be used to reduce mosquito densities [34, 35]. Larvicide treatment is also an alternative method of mosquito control[36]; however, sole reliance on larvicide may not be a long-term solution due to issues of environment concern, cost and resistance development to products used.

For manhole chambers, adoption of completely sealed covers may be the most effective solution to mosquito control; fabrication of a specific removal tool would be required since there would be no pick holes on the cover [22, 24]. Even the absence of surface exit holes did not appear to prevent newly-emerged adult mosquitoes from exiting the underground manholes as reported by Metzger[37], elimination of pick holes in manhole covers does reduce the number of mosquitoes entering and reproducing in these structures [38]. In urban Shanghai, it would be costly to redesign and replace the existing manhole covers with pick holes; taking measures to block these holes would be an alternative solution for mosquito control. Herein we suggested that manhole covers made of plastic composite should be abandoned henceforth, and the existing aging or broken covers should be replaced with cast iron covers for consideration of safety and mosquito control efficiency.

The study has some notable weaknesses. Firstly, besides near-surface drainage system we described here, there was another drainage system located in the subterranean building and constructions in downtown Shanghai, including subterranean parking lots, subway system and so on; the status of mosquito production in such systems is unclear. Secondly, we performed an approximate cross-sectional sampling design for the drainage system during the summer, while less was known about the year-round dynamics of mosquito production. Thirdly, we were not able to conduct physicochemical testing of water samples from different structures, and this made it unclear which types of water were more conducive for production of *Ae*. *albopictus* and other mosquito vectors. Further studies are needed to resolve these questions in the future to improve mosquito control in Shanghai and its surrounding areas.

## Conclusion

The near-surface urban drainage system in urban Shanghai is highly complex, with hundreds of thousands of CBs and MCs. All larvae sampled belonged to two species, *Ae*. *albopictus* and the *Cx*. *pipien* complex, and the former was predominant in both CBs and stormwater MCs, especially in residential neighborhoods. The CBs were the major source of mosquito production in downtown Shanghai, and the vertical-grate type with an unsealed lid was most conducive to larval production. MCs featured more running water and lower percentage of larvae, and few larvae were found in Sewage MCs; however, due to the tremendous baseline amount, MCs were still an important source of mosquito production. We suggested that *Aedes* control in Shanghai should focus more on CBs or other potential larval habitats in and around residential neighborhoods, and that a permeable material and a completely sealed cover should be adopted in construction of CBs and MCs henceforth.

## Abbreviations

CBs: Catch basins
MCs: Manhole chambers
SMCs: Stormwater manhole chambers
Ae. albopictus: *Aedes albopictus*
*Cx*. *pipiens* complex: *Culex pipiens* complex
ANOVA: analysis of variance
